# Continuous Assessment of Left Ventricular Outflow Obstruction in Transcatheter Aortic Valve Implantation

**DOI:** 10.1016/j.jaccas.2025.106523

**Published:** 2025-12-16

**Authors:** Yu Ando, Atsushi Sugiura, Masanori Yamamoto

**Affiliations:** a Department of Cardiovascular Medicine, Nagoya Heart Center, Aichi, Japan; b Department of Cardiovascular Medicine, Toyohashi Heart Center, Aichi, Japan

**Keywords:** hemodynamic monitoring, left ventricular outflow tract obstruction, transcatheter aortic valve implantation, SavvyWire

## Abstract

**Background:**

Left ventricular outflow tract (LVOT) obstruction is a rare but fatal complication after transcatheter aortic valve implantation (TAVI). Early detection and timely intervention are essential for favorable clinical outcomes.

**Case Summary:**

We report a case of LVOT obstruction diagnosed and dynamically monitored via a SavvyWire (Haemonetics). A 78-year-old woman underwent transfemoral TAVI for severe valve stenosis, and continuous hemodynamic monitoring by the SavvyWire identified a significant aorto–left ventricular pressure gradient with systemic hypotension immediately after valve deployment, indicating LVOT obstruction. Prompt administration of intravenous fluids and vasopressors resulted in rapid hemodynamic stabilization.

**Discussion:**

This case underscores the utility of the SavvyWire for immediate detection and management of dynamic LVOT obstruction, potentially improving safety profiles in TAVI procedures.

**Take-Home Message:**

Continuous monitoring of the aorto–left ventricular pressure gradient can facilitate early diagnosis of LVOT obstruction in TAVI procedures.

## Visual Summary

**Figure:**
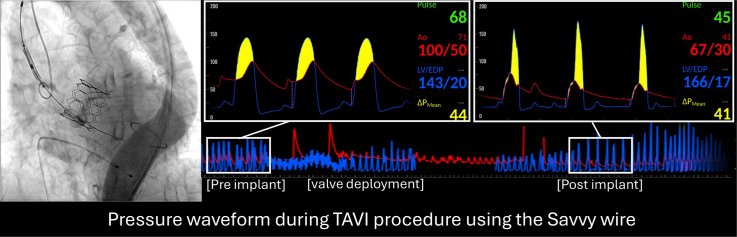

Dynamic left ventricular outflow tract (LVOT) obstruction represents an infrequent but potentially life-threatening complication after transcatheter aortic valve implantation (TAVI) for severe aortic stenosis (AS). Conventional methods often require catheter exchange to assess the aorto–left ventricular (LV) pressure gradient, limiting continuous intraprocedural monitoring capabilities. The SavvyWire (Haemonetics) is a next-generation 0.035-inch support wire for TAVI that combines LV pacing and real-time pressure monitoring in a single device. It enables simultaneous measurement of aortic and LV pressures with high accuracy, correlating with standard catheterization techniques.^1^ Its application during TAVI has been validated for safety and efficacy across diverse valve anatomies.^2^^,^^3^ Its main features are: 1) simultaneous aorto-LV pressure measurement; 2) LV pacing capability; 3) enhanced stiffness; and 4) extended wire length (280 cm). These features enable rapid pacing during valve implantation, simplifying the workflow and eliminating the need for a separate pacing lead. In addition, they enable continuous invasive monitoring of both aortic and left ventricular pressures throughout the procedure without the need for additional catheter manipulation after valve deployment.

We present a case in which the SavvyWire identified a dynamic change in LVOT obstruction immediately after valve implantation, enabling us to detect and manage the LVOT obstruction by applying hydration and vasopressors for hemodynamic stabilization.

## History of Presentation

A 78-year-old woman presented with fatigue and exertional dyspnea. A systolic ejection murmur was heard at the left sternal border of the second intercostal space. Chest x-ray revealed pulmonary congestion, leading to a diagnosis of heart failure due to AS. Transthoracic echocardiography revealed an aortic valve peak velocity of 3.2 m/s, peak pressure gradient of 41 mm Hg, and aortic valve area of 0.62 cm^2^; it additionally demonstrated a lesional but substantial bulge of the septal wall at the level of the LVOT, which was observed without signs of blood flow acceleration at the LVOT or systolic anterior motion of the mitral valve (Video 1). The LV ejection fraction was 69%. Electrocardiography showed a heart rate of 64 beats/min under normal sinus rhythm. Computed tomography exhibited severely calcified aortic valves, the aortic annular perimeter at 75.9 mm, and an annular area of 434 mm^2^. Consistent with the echocardiography images, computed tomography also demonstrated the bulged basal septum, leading to narrowing of the LVOT. The patient was diagnosed with severe AS.

## Investigations, Differential Diagnosis, and Management

**Figure 1:**
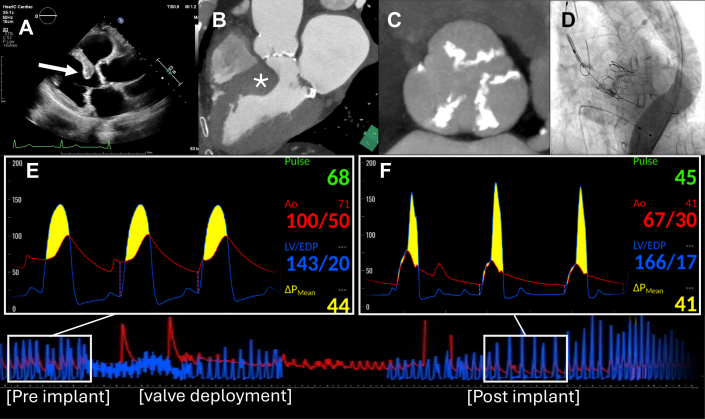
Detecting LVOT Obstruction Using Continuous Pressure Monitoring With the SavvyWire During TAVI (A and B) TTE and CT demonstrating the interventricular septal bulge. (C) All cusps of the aortic valve are severely calcified. (D) TAVI was performed, with the prosthesis delivered using the SavvyWire, while continuously monitoring LV pressure. (E) Before valve implantation, the SavvyWire showed an elevated LV pressure. Aortic pressure exhibited a lower peak compared to LV pressure, confirming the presence of severe aortic stenosis. (F) After valve implantation, a marked drop in aortic pressure was observed, and the SavvyWire revealed a further significant increase in LV pressure and a late systolic peaking of the pressure waveform. These findings indicated acute LVOT obstruction. CT = computed tomography; LV = left ventricle; LVOT = left ventricular outflow tract; TAVI = transcatheter aortic valve implantation; TTE = transthoracic echocardiography.

**Figure 2:**
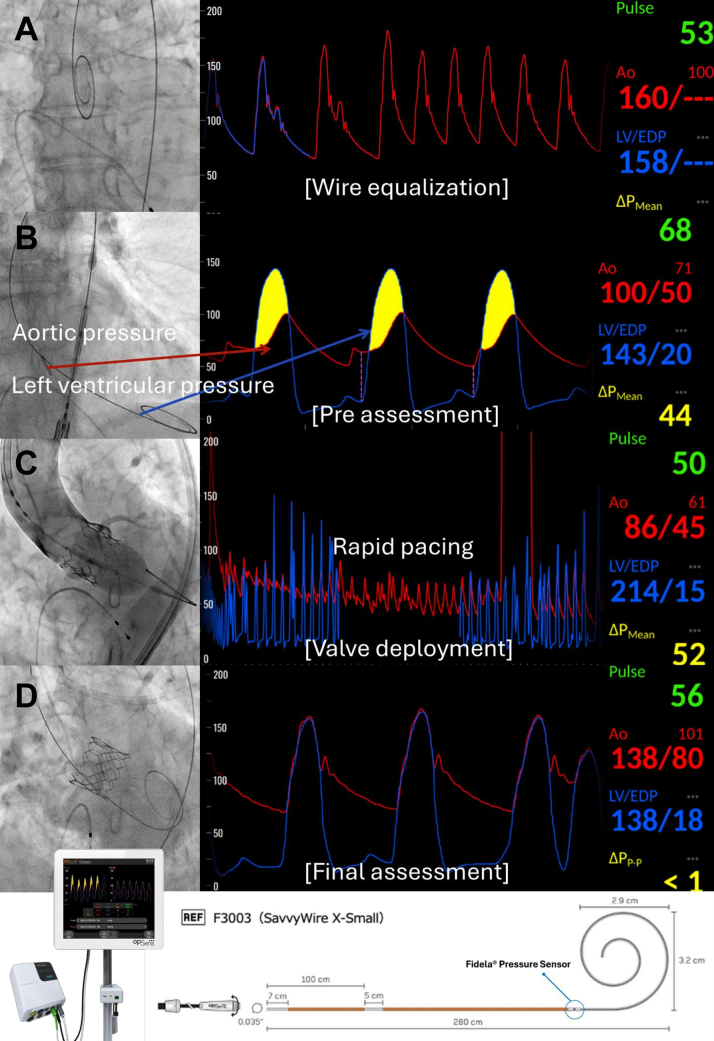
TAVI Workflow Using the SavvyWire (A) First, pressure equalization is performed in the ascending aorta. (B) After the standard TAVI procedure, the SavvyWire is advanced into the LV to allow simultaneous measurement of the aorto-LV pressure gradient. (C) Subsequently, rapid pacing is performed using the SavvyWire during valve deployment. (D) Finally, the aorto-LV pressure gradient is reassessed with the SavvyWire for the final evaluation. In cases where a residual pressure gradient remains, postdilatation is considered. LV = left ventricle; TAVI = transcatheter aortic valve implantation.

Given the age and comorbidities of the patient, the institutional heart team decided to undergo transfemoral aortic valve implantation for severe AS. TAVI was performed using a SavvyWire for delivery system insertion and continuous assessment of LV pressure throughout the procedure, while a pigtail catheter was placed in the ascending aorta for monitoring systemic pressure. Under rapid pacing, a 26-mm Sapien 3 Ultra Resilia prosthesis (Edwards Lifesciences) was deployed at the aortic valve, when the patient immediately developed substantial hypotension, with a systemic blood pressure of 67 mm Hg. Despite multiple potential for shock after balloon-expandable TAVI, continuous hemodynamic monitoring via the SavvyWire revealed an LVOT obstruction, as indicated by the late systolic peaking of the LV pressure waveform and marked pressure gradient between the LV and ascending aorta of 41 mm Hg (Figure 1). Therapeutic measures, including rapid fluid resuscitation and administration of phenylephrine hydrochloride, resulted in prompt hemodynamic stabilization and resolution of the pressure gradient. Eventually, the peak-to-peak aorto-LV pressure gradient completely disappeared, confirming the resolution of dynamic LVOT obstruction (Figure 2). Additionally, other differential diagnoses for hypotension, such as cardiac tamponade, paravalvular leakage, or coronary obstruction, were excluded by echocardiography and angiography.

## Outcome and Follow-Up

During hospitalization, the patient exhibited no signs of hemodynamic instability or worsening heart failure. She was discharged without complications.

## Discussion

Dynamic LVOT obstruction is typically caused by an acute decline in LV afterload, followed by a hyperkinetic LV wall. Although rare, this condition can be fatal if timely recognition and intervention are not achieved. Most importantly, inotropic agents (eg, dobutamine, adrenaline) may worsen the obstruction. Several factors are known to be associated with LVOT obstruction after surgical aortic valve replacement, including LV septal hypertrophy, a sigmoid-shaped septum, and a narrow LVOT at baseline.^4^ Early recognition is fundamental in the case of LVOT obstruction after TAVI.

The SavvyWire provides continuous, real-time monitoring of the aorto-LV pressure gradient after valve deployment, facilitating early diagnosis of LVOT obstruction or residual pressure gradient through the prosthetic valve. It guides diagnostic work-up and therapeutic decisions in cases of hypotension after valve implantation.^2^ In contrast, conventional techniques necessitate wire exchange for pigtail catheter insertion into the LV to measure the pressure gradient, which risks hemodynamic instability. The SavvyWire streamlines this process, allowing continuous assessment of pressure gradient throughout valve deployment. We also consider that evaluating the aorto-LV waveform using the SavvyWire after TAVI may help detect the presence of paravalvular leak. However, further studies are warranted to validate its efficacy in assessing aortic regurgitation or paravalvular leak after valve implantation.

## Conclusions

This case demonstrates the clinical value of real-time hemodynamic monitoring using the SavvyWire in patients undergoing TAVI. Early recognition and prompt management with volume administration and vasopressors are critical for hemodynamic stabilization. This report highlights the clinical utility and safety profile of the SavvyWire, emphasizing its role in enhancing procedural safety.

**Visual Summary:**
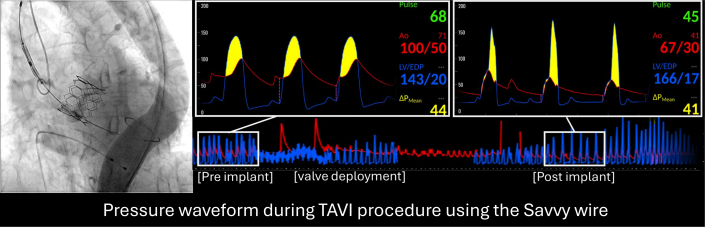
Pressure Waveform During TAVI Using the SavvyWire The SavvyWire is a next-generation guidewire that enables both rapid pacing and continuous real-time aorto-LV pressure monitoring. This case report describes a patient who developed dynamic LVOT obstruction immediately after valve implantation, in whom continuous assessment of the aorto-LV pressure gradient facilitated prompt diagnosis and treatment. LV = left ventricular; LVOT = left ventricular outflow tract; TAVI = transcatheter aortic valve implantation.

## Funding Support and Author Disclosures

The authors have reported that they have no relationships relevant to the contents of this paper to disclose.

**Take-Home Messages**

• Continuous real-time monitoring of the aorto-LV pressure gradient with the SavvyWire during TAVI enables early detection of LVOT obstruction.
• Timely volume administration and vasopressor therapy can rapidly reverse dynamic LVOT obstruction post-TAVI.

## Appendix

Video 1 Pre-TAVI Echocardiographic Findings of Basal Septal Bulge Transthoracic echocardiography demonstrates a basal interventricular septal bulge at the LVOT without flow acceleration or systolic anterior motion of the mitral valve. LVOT = left ventricular outflow tract; TAVI = transcatheter aortic valve implantation.
